# Morphological changes and luminescence of *Escherichia coli* in contact with Mn_2_O_3_ and Co_3_O_4_ ultrafine particles as components of a mineral feed additive

**DOI:** 10.14202/vetworld.2024.1880-1888

**Published:** 2024-08-24

**Authors:** Daniil Evgenievich Shoshin, Elena Anatolievna Sizova, Aina Maratovna Kamirova

**Affiliations:** 1 Federal Research Centre of Biological Systems and Agrotechnologies of the Russian Academy of Sciences, Orenburg, Russia; 2 Federal State Budgetary Educational Institution of Higher Education Orenburg State University, Orenburg, Russia

**Keywords:** atomic force microscopy, cell wall, cobalt, *Escherichia coli*, luminescence, manganese, nanotechnology, ultrafine particles

## Abstract

**Background and Aim:**

The spread of antibiotic resistance and mineral depletion in soils encourages an intensive search for highly effective and environmentally safe bactericidal agents and sources of macro- and micro-elements. The most profitable solution would combine both the described tasks. Ultrafine particles (UFPs) have this functionality. Thus, this study aimed to analyze the bioluminescence and external morphological changes of *Escherichia coli* cells after contact with M_2_O_3_ and Co_3_O_4_ UFPs at effective concentrations (ECs).

**Materials and Methods:**

The antibiotic properties of the studied samples were determined on a multifunctional microplate analyzer TECAN Infinite F200 (Tecan Austria GmbH, Austria) by fixing the luminescence value of the bacterial strain *E. coli K12 TG11* (Ecolum, NVO Immunotech Closed Joint Stock Company, Russia). Morphological changes in the cell structure were evaluated using a Certus Standard EG-5000 atomic force microscope equipped with NSPEC software (Nano Scan Technology LLC, Russia).

**Results:**

The obtained results indicate high bactericidal properties of Co_3_O_4_ and Mn_2_O_3_ UFPs (EC_50_ at 3.1 × 10^−5^ and 1.9 × 10^−3^ mol/L, respectively) due to the degradation of the cell wall, pathological increase in size, disruption of septic processes, and loss of cytoplasmic contents.

**Conclusion:**

The prospects for the environmentally safe use of ultrafine materials are outlined. The limits of the dosages of Co_3_O_4_ and Mn_2_O_3_ UFPs recommended for further study *in vitro* and *in vivo* in feeding farm animals are established (no more than 4.9 × 10^−4^ mol/L for Mn_2_O_3_ UFPs and 1.5 × 10^−5^ mol/L for Co_3_O_4_ UFPs). The limitation of the work is the lack of experiments to determine the mechanisms of the toxic effect of UFP on bacteria, protein structures, and DNA and oxidative stress, which is planned to be performed in the future together with *in situ* and *in vivo* studies on animals.

## Introduction

Among the problems existing today in the livestock industry and directly related to nutrition processes, special attention should be paid to antibiotic resistance and the mineral value of feed resources because both of these issues directly affect the productivity and efficiency of production in general [1, 2].

Previously, widely used antibiotic agents contributed to the growth of live weight due to the suppression of pathogenic microflora and general modulation of the species composition of the gastrointestinal tract microbiota. Animals receive the necessary macro and microelements from feeding on pastures. Today, there are concerns about the developing resistance of microorganisms and the possibility of their global spread through food [3, 4]. Mineral depletion of soils is also noted, which results in a lack of essential elements in coarse, succulent, and green feeds [5].

This makes it extremely urgent to search for safe alternatives to antibiotics for growth stimulants and highly effective sources of macro- and microelements, especially against the background of the prohibitions imposed on antibiotics. The sources of macro- and microelements are especially important because, despite their low demand, they play a significant role in metabolic processes. They transport oxygen and carbon dioxide, are part of vitamins, hormones, and enzymes, maintain pH and osmotic balance, and neutralize toxins [6, 7].

A positive solution to this problem is the combination of two functions, bactericidal and nutritional. Ultrafine particles (UFPs) and nano- and micro-sized composites of various compositions, including metallic forms of chemical elements, have this functionality. They not only suppress pathogenic microorganisms, as shown by the example of silver, iron, zinc, and copper [8, 9], but also act as a source of the corresponding macro- or microelement in the animal’s diet [10].

However, their application has some limitations due to the significant reactivity due to the small diameter and high ratio of surface area to volume [11]. Thus, before the introduction of UFPs into the feed, preliminary certification of biological effects *in vitro* should be performed to establish subinhibitory dosages and exclude possible negative effects on the host body.

Thus, the research problem of the presented work is to find and certify alternatives to antibiotic growth stimulants with a potential supplemented effect in the form of a source of trace elements in the diet.

Thus, this study aimed to analyze the bioluminescence and external morphological changes of *Escherichia coli 25322 cells* after contact with Mn_2_O_3_ and Co_3_O_4_ UFPs at effective concentrations (EC). For the first time, as far as the authors know, this will allow us to directly correlate ECs with morphological changes in the structure of bacterial cells and identify dosages recommended for further testing on animals.

## Materials and Methods

### Ethical approval

The ethical approval is not applicable. The study does not contain experiments on animals or humans. The study is conducted on bacterial strains that do not belong to the group of pathogenic ones.

### Study period and location

The study was conducted in October 2023 in three stages at the Nanotechnology in Agriculture Center of the Federal State Budgetary Research Institution (FSBRI) “Federal Research Center of Biological Systems and Technology” (FRC BST) of the Russian Academy of Sciences (RAS), Orenburg, Russia.

### Experimental design

#### First stage

Determination of the UFP diameter and Z potential using dynamic light scattering (DLS): Co_3_O_4_ and Mn_2_O_3_ UFP samples of 20.1 and 19.8 mg were suspended in 1 mL of distilled water with ultrasound at a frequency of 35 kHz for 30 min (Sapphire 4.0 ultrasonic bath, Russia), after which in the amount of 500 μL, they were put into the cuvette of the Microtrac NANOTRAC WAVE II laser analyzer (Microtrac, USA), where the corresponding indicators were recorded.

#### Second stage

Identification of EC suppressing 80, 50, and 20% luminescence of the *E. coli K12 TG1* bacterial strain carrying a hybrid plasmid *pUC19* with cloned *luxCDABE* genes *Photobacterium leiognathi 54D10* (commercial name Ecolum, NVO IMMUNOTECH, Russia) using a TECAN Infinite F200 multifunctional microplate analyzer (Tecan Austria GmbH, Austria).

To achieve this, Co_3_O_4_ and Mn_2_O_3_ UFP samples of 40.1 and 39.5 mg were suspended in 1 mL of distilled water according to the same algorithm as in the first stage. In a 96-well culture plate, a series of step dilutions (100 μL/cell) of Co_3_O_4_ and Mn_2_O_3_ were prepared at a dose from 5.0 × 10^−1^ to 6.0 × 10^−8^ mol/L, expressed as cobalt and manganese. The Ecolum bacterial test system was prepared in advance: 5 mL of distilled water cooled to 4°C and the same amount of room temperature water were poured into the lyophilized strain, intensively shaken, and kept in the refrigerator for 30 min, after which 100 mL was added to the cells of the UFPs under study. As a result, we obtained concentrations from 2.5 × 10^−1^ to 3.0 × 10^−8^ mol/L. The plate was loaded into the device, and the intensity of the glow was recorded for 3 h with an interval of 5 min.

Based on the data obtained, tables reflecting the dynamics of glow inhibition were constructed, and the relative value of bioluminescence was calculated using the following formula:

A=Io/Ik×100%

where *Ik* is the luminosity of the control sample

where *Io* is the luminosity of the experimental sample.

The effectiveness of the method has been proven by the example of *Vibrio fischeri* and *E. coli* strains in the analysis of wastewater toxicity [12] and bactericidal activity of ultrafine metal particles [13].

#### Third stage

Diagnostics of external morphological changes in the relief of the museum *E. coli 25322* bacterial strain after contact with the substances under study were carried out on a Certus Standard EG-5000 atomic force microscope with NSPEC software (Nano Scan Technology Limited Liability Company [LLC], Russia). To do this, a daily culture of bacteria grown on Luria–Bertani (LB) agar was suspended in LB broth to an optical density 0.2 units higher than the background value at a wavelength of 450 nm and poured into a 96-well plate by 100 μL/cell, after which 100 μL of the test sample was added. The plate was covered with a lid and placed in a thermostatically controlled shaker (ELMI, Latvia) at 37°C and 300 rpm for growth. The optical density was recorded every 30 min until it reached 0.5 units at 450 nm above the background in the control cells (100 μL of distilled water), which corresponds to the stationary phase of culture growth. The samples were placed in 1 mL Eppendorf and centrifuged at 3000×*g* for 2 min. The filler liquid was then removed and 500 mL of distilled water was poured, bringing the optical density to 0.1–0.2 units above the background. The resulting suspensions were kept on a vortex V-32 (BioSan, Latvia) for 20–30 s to separate the cells and applied to sterile cover glasses without chips and roughness in a volume of 10 μL. They were dried at room temperature (22°C–24°C) and then scanned in intermittent contact, obtaining a 3D image of the surface relief of the bacterial cells [14].

### Statistical analysis

The reliability of the differences between the absolute values of luminescence and the dimensional characteristics of bacteria was determined using Student’s t-test with a required significance level of p ≤ 0.01. The tables show the relative values corresponding to the presented threshold.

## Results

The diameter of Mn_2_O_3_ and Co_3_O_4_ UFPs determined using the DLS method was 918.3 ± 55.5 and 880.1 ± 24.8 nm, respectively, with a Z potential of 22.2 ± 1.3 and 23.9 ± 2.2 mV.

When *E. coli K12 TG1* suspension was contaminated with Co_3_O_4_ UFPs in the range from 2.5 × 10^−1^ to 6.1 × 10^−5^ mol/L, absolute suppression of luminescence was observed in the last minutes of exposure (p ≤ 0.001), while the luminosity of the bacterial strain at the beginning of the experiment was higher when less Co_3_O_4_ UFPs were introduced into the medium; the value varied from 0.24% to 50.55% compared with the control variant, after which these indicators fell sharply to 14.51% and 0.22% of the original value, respectively. In 90–120 min at a dose of 9.8 × 10^−4^ mol/L, a short-term increase in raw luminescence units (RLU) was observed as a sign of indifferent colony-forming units. The labile zone of the biochemical transition included concentrations of 6.1 × 10^−5^ (EC_80_), 3.1 × 10^−5^ (EC_50_), and 1.5 × 10^−5^ (EC_20_) mol/L (Table-1).

**Table-1:** Relative luminescence values of *Escherichia coli* K12 TG1 in a medium with different Co_3_O_4_ UFP content.

Time (min)	Concentration (mol/L)											

	7.8×10^−3^	3.9×10^−3^	1.9×10^−3^	9.8×10^−4^	4.9×10^−4^	2.4×10^−4^	1.2×10^−4^	6.1×10^−5^	3.1×10^−5^	1.5×10^−5^	7.6×10^−6^	3.8×10^−6^
0	4.62	7.22	10.56	13.76	17.73	27.71	36.28	50.55	71.45	88.46	110.7	127.8
30	0.05	0.05	0.05	0.05	0.04	0.24	1.32	9.62	40.39	66.54	89.74	111.5
60	0.03	0.04	0.05	0.05	0.05	0.05	0.07	1.44	23.51	56.98	84.58	105.9
90	0.05	0.04	0.05	0.05	0.05	0.05	0.05	0.28	19.58	59.54	92.29	110.0
120	0.03	0.04	0.04	0.05	0.05	0.05	0.04	0.11	21.28	71.08	97.71	110.9
150	0.03	0.04	0.04	0.05	0.06	0.05	0.04	0.06	24.18	76.86	85.18	96.27
180	0.03	0.04	0.05	0.05	0.05	0.06	0.04	0.04	24.92	70.13	79.62	90.92
Average value	0.69	1.07	1.55	2.01	2.58	4.03	5.41	8.87	32.19	69.94	91.40	107.6

The numerical data correspond to the value of the relative luminescence value A (%). The color fill corresponds to the following indicators: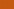: toxic (Tox),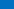 : EC_80_,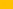 :EC_50_,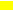 :EC_20_,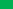 : non-toxic (NTOX), that is, UFP concentrations causing over 95, 80, 50, and 20% of biosensor quenching and having no negative effect. UFP=Ultrafine particles, EC: Effective concentration

Furthermore, the introduction of Co_3_O_4_ UFPs was accompanied by a short-term increase in bioluminescence by 10.71 (p ≤ 0.01) and 27.83% (p ≤ 0.001). In the first case (7.6 × 10^−6^ mol/L), this effect persisted for no more than 30 min, and in the second case (3.8 × 10^−6^mol/L), it persisted for 120 min. In turn, the introduction of Mn_2_O_3_ UFPs into the nutrient medium had a significantly less bactericidal effect. No doses inhibiting the glow were detected (Table-2).

**Table-2:** Relative luminescence values of *Escherichia coli* *K12 TG1* in a medium with different Mn_2_O_3_ UFP content.

Time (min)	Concentration (mol/L)										

	2.5×10^−1^	1.2×10^−1^	6.2×10^−2^	3.1×10^−2^	1.6×10^−2^	7.8×10^−3^	3.9×10^−3^	1.9×10^−3^	9.8×10^−4^	4.9×10^−4^	2.4×10^−4^
0	20.98	21.31	20.57	23.01	27.20	33.08	42.23	56.69	75.65	84.26	91.70
30	19.62	19.66	20.61	24.23	26.55	31.65	39.11	53.00	70.50	76.00	84.32
60	16.15	17.83	21.49	26.14	27.17	32.33	41.55	55.66	74.62	80.12	89.15
90	17.15	18.43	22.75	24.97	26.77	31.27	38.95	54.64	75.61	84.28	94.42
120	18.51	19.22	20.79	22.84	24.60	28.69	35.71	50.85	71.57	81.91	93.92
150	19.57	19.65	18.67	20.53	23.02	26.77	33.64	48.56	68.78	79.33	92.34
180	19.87	19.09	17.00	18.23	21.28	25.46	32.72	46.68	65.87	75.93	89.85
Average value	18.84	19.31	20.27	22.85	25.23	29.89	37.70	52.30	71.80	80.26	90.81

The numerical data correspond to the value of the relative luminescence value A (%). The color fill corresponds to the following indicators: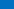 : EC_80_,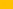 : EC_50_,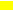 : EC_20_,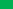 : and NTOX, that is, UFP concentrations causing over 95, 80, 50, and 20% of biosensor quenching and having no negative effect. UFP=Ultrafine particles, EC: Effective concentration

Concentrations from 2.5 × 10^−1^ to 6.2 × 10^−2^ mol/L suppressed over 80% of luminescence, from 3.1 × 10^−2^ to 1.9 × 10^−3^ over 50%, and from 9.8 × 10^−4^ to 4.9 × 10^−4^ over 20% (p ≤ 0.001). Mn_2_O_3_ UFPs at a dose of 3.1 × 10^−5^ mol/L stimulated the glow of *E. coli K12 TG1* in the range from 3.74% to 14.04% compared with the control variant (p ≤ 0.05). Consequently, they are 4000 times less toxic than Co_3_O_4_ UFPs and are characterized by a much wider zone of biochemical transition (nine double dilutions vs. three).

The ratio of the RLU value at the end of the experiment to the same value at the 1^st^ min ranged from 88.82% to 120.12%, depending on the concentration, which indicates a stable bacteriostatic effect in contrast to the prolonged antibiotic action of Co_3_O_4_ UFPs.

The assessment of surface changes in the structure of bacterial membranes performed using atomic force microscopy showed that the level of damage directly depended on the concentration of Co_3_O_4_ and Mn_2_O_3_ UFPs (Table-3), and the former acted more aggressively.

**Table-3:** The proportion of damaged *Escherichia coli* *25322* cells during contamination of the suspension with Co_3_O_4_ and Mn_2_O_3_ UFPs with different levels of toxicity.

Type of morphological disorder	UFP Co_3_O_4_		

	EC_80_	EC_50_	EC_20_
Increase in size	-	3/15	2/15
Formation of protrusions	4/15	2/15	2/15
Disruption of septic processes	-	1/15	-
Loss of cytoplasmic contents	9/15	3/15	-

**Type of morphological disorder**	**UFP Mn_2_O_3_**		

	**EC_80_**	**EC_50_**	**EC_20_**

Increase in size	1/15	1/15	1/15
Formation of protrusions	4/15	3/15	2/15
Disruption of septic processes	1/15	2/15	-
Loss of cytoplasmic contents	6/15	1/15	-

UFP=Ultrafine particles, EC: Effective concentration

**Figure-1:**
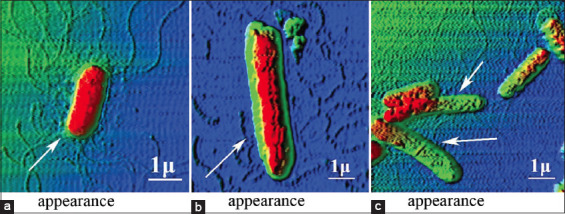
Increase in the size of *Escherichia coli* *25322* with UFP contamination. a=Control, b=Co_3_O_4_ (EC_50_), c=Mn_2_O_3_ (EC_50_). The cell sizes are indicated in μm. UFP=Ultrafine particles, EC: Effective concentration.

**Figure-2:**
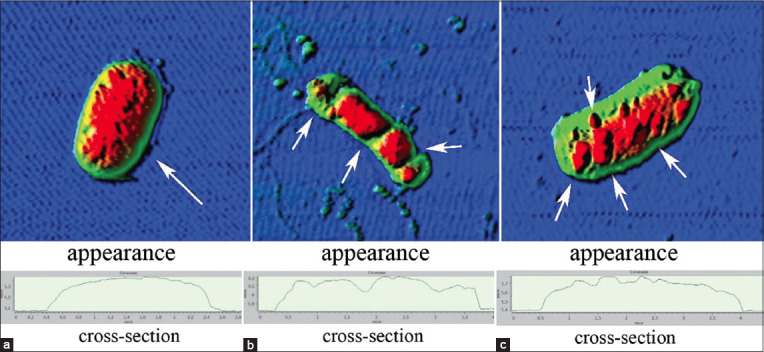
Protrusions of the *Escherichia coli* *25322* cell wall with UFP contamination. a=Control, b=General view and cross-section for Co_3_O_4_ (EC_50_), and c=General view and cross-section for Mn_2_O_3_ (EC_50_). UFP=Ultrafine particles, EC: Effective concentration.

**Figure-3:**
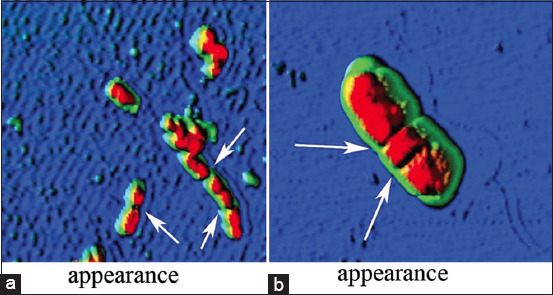
Disruption of the septic processes of *Escherichia coli* *25322* with UFP contamination. a=Mn_2_O_3_ (EC_50_) and b=Co_3_O_4_ (EC_50_). UFP=Ultrafine particles, EC: Effective concentration.

**Figure-4:**
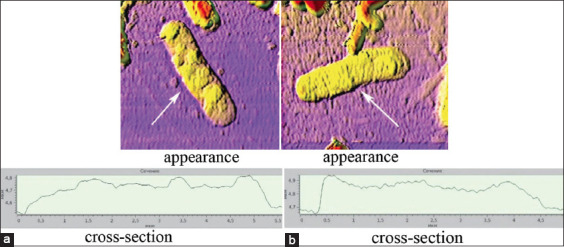
Loss of cytoplasmic contents of *Escherichia coli* *25322* with UFP contamination a=Mn_2_O_3_ (EC_80_) and b=Co_3_O_4_ (EC_80_). UFP=Ultrafine particles, EC: Effective concentration.

However, the nature of the damage in both cases was heterogeneous, including a non-systemic increase in size from 2.3 ± 0.1 to 4.2 ± 0.2 μm (p ≤ 0.001) (Figure-1), i.e., by 82.6%, the formation of cell wall protrusions of up to 42.1% of the total height (Figure-2), disruption of septic processes and the appearance of filamentous forms from two to four sections (Figure-3), and loss of cytoplasmic contents (Figure-4).

**Figure-5:**
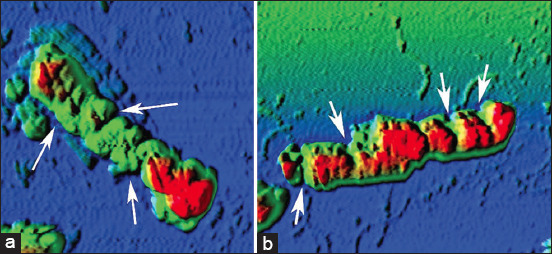
Destroyed cells of *Escherichia coli* *25322*. a=In a medium with Co_3_O_4_ UFPs (EC_80_) and b=In a medium with Mn_2_O_3_UFPs (EC_80_). UFP=Ultrafine particles, EC: Effective concentration.

The latter was more characteristic of high levels of UFP toxicity. Protrusions were observed in all experimental samples and a minor form was observed in the control variant. All degenerative processes together led to complete lysis of the bacterial cells (Figure-5).

Thus, Co_3_O_4_ and Mn_2_O_3_ UFPs in doses over 3.1 ×10^−5^ and 1.9 × 10^−3^ have a pronounced bactericidal effect, completely inhibiting the vital activity of *E. coli*.

## Discussion

Changes in metabolic activity and degenerative disorders in the external structure of bacteria after contact with manganese- and cobalt-containing UFPs prove the effectiveness of the latter as an alternative to antibiotic agents and are consistent with earlier studies. It has been shown that the UFPs under study not only inhibit the growth and reproduction of *E. coli* against the background of membrane degradation and oxidative stress [15, 16] but also actively inhibit the nitrification processes in a bioreactor (7%–10%) and *in vivo*, simultaneously reducing the transcription levels of three key functional genes, *amoA, hao*, and *nirK*, involved in the redox transformations of nitrogen [17]. For example, Co_3_O_4_ UFPs reduced the total number of prokaryotes and, in particular, the abundance of representatives of the genus *Azotobacter*, the activity of catalase, and the dehydrogenase of ordinary chernozem [18].

The changes in the external structure of *E. coli* detected by atomic force microscopy replicate similar manifestations when exposed to the antibiotic ampicillin [14]. We suggest that the increase in cell size may be due to the effect of internal osmotic pressure on the cell wall, which has decreased its rigidity.

Moreover, CoO and MnO UFPs were effective against *Candida albicans* mold fungi [19] and *Saccharomyces cerevisiae* yeast [20], reducing oxygen consumption by the latter (50% at 170 mg/L) and causing damage to the cytoplasmic membrane (≈30% of cells at 1000 mg/L). Similarly, Mn_2_O_3_ UFPs provoked the apoptosis of *Leishmania* major promastigote (57% at 15 μg/mL) both *in vitro* and *in vivo*, increasing the survival rate of infected mice [21]. They also acted as analogs of pyrimethamine and sulfadiazine, inhibiting the growth of *Toxoplasma gondii* tachyzoite (40% at 105 μg/mL) [22].

Thus, it can be concluded that UFPs provoke system-wide disorders in the work of cells, which, as a rule, are associated with several mechanisms:

- Direct association (co-agglomeration) with the cytoplasmic membrane, its damage, initiation of internal signaling pathways leading to death, or isolation from nutrients and light energy
- Dissolution of UFPs and release of toxic ions that disrupt important enzyme functions or interact directly with the DNA
- Formation of reactive oxygen species (ROS) and subsequent oxidative stress in the body [23].

For example, it has been reported by Wang *et al*. [24] that the inhibitory effect of ZnO UFPs on *Photobacterium phosphoreum* bioluminescence was solely due to the leaching of bioavailable Zn^2+^ ions, whereas the bactericidal effects of CuO UFPs were associated with both ion production and direct exposure to particles. UFP action was also the main cause of the toxicity of Fe_2_O_3_, Co_3_O_4_, Cr_2_O_3_, and NiO [24]. Concerning the TiO_2_ UFP, it has been shown that they actively bound to the membrane of *Raphidocelis subcapitata*, blocking access to light rays, similar to Pd, CuO, Co_3_O_4_, TiO_2_, Mn_3_O_4_, and Fe_3_O_4_ UFPs [25]. Similar effects were found in other *Scenedesmus* spp. and *Chlorella* spp. algae [26]. UFPs can also adsorb nutrients on their surface, reducing their concentration in the environment, as shown by the example of CeO_2_, which absorbs phosphates, iron, and molybdenum ions [27]. However, ROS generation is still of the greatest importance, especially as a result of UFP photoactivation [25].

Considering all of the above, one should separately note the possibility of modulating the bactericidal activity of UFP, depending on a set of parameters, such as surface charge, shape, material, concentration, degree of dispersion, solubility, relationship with the components of the medium and pH, physicochemical properties, specific ratio of surface area to volume, size, differences in the structure of the bacterial cell wall, and the effect of ultraviolet illumination [28].

### Physicochemical nature

To illustrate, in a 24-h point test to study 12 metal-containing UFPs against *E. coli* and *Staphylococcus aureus*, Co_3_O_4_ and Mn_3_O_4_ UFPs along with Fe_3_O_4_ UFPs (minimum bactericidal concentration: 100 mg/L) occupied an intermediate position between CuO (1 and 0.1 mg/L), ZnO (10 mg/L), Pd (10 and 100 mg/L) and Al_2_O_3_, MgO, Sb_2_O_3_, SiO_2_, TiO_2_, and WO_3_ UFPs (not toxic at a dose of <100 mg/L) [25]. It was also found that the cytotoxic response strongly correlated with changes in the physicochemical and electrochemical properties of surface oxides of metallic UFPs, the most powerful of which were Cu UFPs [29].

### Duration of action

In an experiment with CaCo_2_ and A549 cell lines, it was shown that Sb_2_O_3_, Mn_3_O_4_, and TiO_2_ UFPs did not have a toxic effect for 24 h at a dose of 100 μg/mL or less, whereas Co_3_O_4_ and ZnO UFPs had a moderate negative effect and CuO UFPs were toxic even at low concentrations (24 h, EC_25_ = 11 for A549 and 71 μg/mL for Caco_2_). However, with longer exposure (up to 9 days), the toxic effects of Mn_3_O_4_ and Sb_2_O_3_ increased significantly (the value in case of 9 days, EC_50_ for A549 and Caco_2_ for Sb_2_O_3_ was 22 and 48 μg/mL and for Mn_3_O_4_ 47 and 29 μg/mL, respectively) [30].

### Mineral composition

The MnCoO nanocomposite showed an enhanced dose-dependent insecticidal effect on *Culex pipiens* mosquito larvae and pupae, causing 100% death compared with Mn_2_O_3_ UFPs, which had a mortality rate of 88% at high concentrations [31]. In addition, Kainat *et al*. [32] found that Co_3_O_4_ UFPs had larvicidal activity against *Aedes aegypti* with a mortality rate of 67.2%.

### Size

In another study by Singh *et al*. [33], 28-day oral toxicity, genotoxicity, biochemical and histopathological changes, and distribution of nano-UFPs (nUFPs) and micro-sized UFPs (mUFPs) of manganese oxide (MnO_2_) in tissues were evaluated in Wistar rats. The results demonstrated a significant increase in DNA damage in leukocytes and micronuclei as well as the number of chromosomal aberrations in bone marrow cells after exposure to MnO_2_ nUFPs at doses of 1000 and 300 mg/kg live weight per day and MnO_2_ mUFPs at a dose of 1000 mg/kg. Acetylcholinesterase inhibition was detected at 1000 and 300 mg/kg/day in the blood and at all doses in the brain. In addition, the activities of aspartate aminotransferase, alanine aminotransferase, and lactate dehydrogenase in the liver, kidneys, and blood serum were significantly suppressed in a dose-dependent manner. MnO_2_ nUFPs showed a much higher absorption capacity and distribution in tissues than MnO_2_ mUFPs. Histopathological analysis showed that MnO_2_ nUFPs caused changes in the liver, spleen, kidneys, and brain. In other words, MnO_2_ nUFPs induced toxicity at lower doses than MnO_2_ mUFPs [33].

Similar size-dependent effects were found in the intratracheal instillation of MnO_2_ UFPs (9.14 ± 1.98, 42.36 ± 8.06, and 118.31 ± 25.37 nm) in rats for 6 weeks at doses of 3 and 6 mg/kg of body weight [34].

### Subject of action

The degree of inhibition of the metabolic functions of prokaryotes also depends on their morphological features. For example, Gram-positive *Staphylococcus* was more susceptible to the action of Mn_3_O_4_ UFPs than Gram-negative *E. coli*, whereas Co_3_O_4_ UFPs had the opposite effect [25].

### Additional processing

Various manipulations performed with UFPs (the so-called redesign), including changing the shape, surface charge, and surface area to volume ratio, adding ligands, chelating agents, or antioxidants, and legation, make it possible to change their biological activity in one direction or another [23]. In particular, the addition of embryonic bovine serum to a medium containing MnO and NiO UFPs reduced the rate of their agglomeration and precipitation and increased their solubility [35]. Similarly, the introduction of a dispersant contributed to the inhibition of the bactericidal activity of MnO UFPs at low doses [23].

### Concentration

This is perhaps the most important parameter because one needs to have an accurate idea of the dose of certain UFPs that should be administered to animals to suppress pathogenic microflora without affecting the host cells. Neglecting this threshold can lead to extremely undesirable consequences. Thus, for example, it was found that MnO UFPs with an average diameter of 18.4 ± 5.4 nm, prepared by laser ablation and administered to rats at a dose of 0.50 or 0.25 mg 3 times a week for up to 18 injections, led to disturbances in the neurons of the caudate nucleus and hippocampus [36]. In another experiment, MnO_2_ UFPs caused a significant decrease in the number of spermatozoa, spermatogonia, and spermatocytes, the diameter of the seminal tubes (p ≤ 0.001), and sperm motility. However, there was no significant difference in the weight of the prostate gland, appendage of the testicle, or estradiol and testosterone [37].

The same significance of the concentration was noted in the experiment to determine the toxicological effects of Co UFPs on six different cell lines representing the lungs, liver, kidneys, intestines, and immune system. The second and third places here were given, respectively, to the type of compound (UFP, soluble salts) or the type of cell model and exposure time [38].

Despite the aforementioned concerns, UFPs are still less toxic than their ionic counterparts. Thus, cobalt given with food to *Drosophila melanogaster* larvae in concentrations from 0.1 to 10 mM in ionic form was significantly more genotoxic than Co UFPs, which was expressed in a greater number of mutant spots on the wings of adult flies [39].

Similarly, Co_3_O_4_ and Mn_2_O_3_ UFPs with a diameter of 10–30 nm were toxic to *Daphnia magna* at EC_50_ >100 mg metal/L, whereas the corresponding values for soluble salts were 3.2 mg Co/L and 41 mg Mn/L [40].

Moreover, UFPs have several advantages, such as active binding to biological substrates; prolongation of the time spent in the gastrointestinal tract; effective delivery of the necessary components to the target areas; minimization of pressure from intestinal clearance; rapid absorption by mucosal cells; induction of fenestration of the epithelial lining, for example, the liver; penetration into deep tissues through the capillary bed; uniform distribution and release of minerals, as well as specific accumulation at the inflammation sites [41].

In addition, as already noted, the processes of UFP distribution (absorption, metabolic pathways, excretion) can be modulated depending on their physicochemical characteristics (solubility, size, etc.). For example, UFPs with a diameter of <300 nm dissociate with the bloodstream, and those with a diameter of <100 nm penetrate organs and tissues directly [42]. Another feature is that when UFPs enter the blood, mucous secretions, lymph, and intestinal juices, they are instantly covered with proteins, amino acids, sugars, etc., acquiring a specific biological identity. In addition, nanoadditives can be integrated with micelles or capsules of protein or any natural feed ingredient [43].

When using UFPs in animal husbandry, they are expected to have characteristics such as high low-dose power, better bioavailability, and stable interaction with other compounds [44, 45]. Some UFPs improve the health, immune status, functioning of the digestive system, microbiota homeostasis, metabolism, and reproductive performance of ruminants [46]; in particular, they affect rumen fermentation and increase live weight. Thus, UFPs are an alternative to feed antibiotics in sub-inhibitory doses [47] and contribute to producing safe animal products [48].

## Conclusion

Highly functional UFP preparations of metallic nature based on cobalt and manganese exhibit pronounced bactericidal activity against various strains of *E. coli*, not only disrupting the course of metabolic processes, which is confirmed in the luminescence inhibition test, but also contributing to the degradation of to varying degrees of the cell wall.

The minimum inhibitory concentrations for UFP Co_3_O_4_ and Mn_2_O_3_ were 4.9 × 10^−4^ and 1.5 × 10^−5^ mol/L, respectively. At the same time, the following changes in cell morphology were characteristic for each EC: for EC_20_ – without pronounced changes; EC_50_ – violation of the cell wall surface relief and septic processes, pathological change in cell size; EC_80_ – loss of cytoplasmic contents and cell destruction. Thus, the presented UFPs can be recommended in sub-inhibitory doses as an alternative to antibiotic drugs in animal husbandry. The limitation of the work is the lack of experiments to determine the mechanisms of the toxic effect of UFP on bacteria: on protein structures, DNA and oxidative stress, which is planned to be performed in the future together with *in situ* and *in vivo* studies on animals.

## Data Availability

The datasets generated during the current study are available from the corresponding author on reasonable request.

## Authors’ Contributions

DES, EAS, and AMK: Conceptualized and designed the study and prepared the materials and data collection and analysis. DES: Drafted the manuscript. All authors have read, reviewed, and approved the final manuscript.

## References

[1] Cheng G, Hao H, Xie S, Wang X, Dai M, Huang L, Yuan Z (2014). Antibiotic alternatives:The substitution of antibiotics in animal husbandry?. Front. Microbiol.

[2] Zampiga M, Calini F, Sirri F (2021). Importance of feed efficiency for sustainable intensification of chicken meat production:Implications and role for amino acids, feed enzymes and organic trace minerals. Worlds Poult. Sci. J.

[3] Panin A.N, Komarov A.A, Kulikovsky A.V, Makarov D.A (2017). Problem of antimicrobial resistance of zoonotic bacteria. Vet. Med. Anim. Sci. Biotechnol.

[4] Low C.X, Tan L.T.H, Ab Mutalib N.S, Pusparajah P, Goh B.H, Chan K.G, Lee L.H (2021). Unveiling the impact of antibiotics and alternative methods for animal husbandry:A review. Antibiotics (Basel).

[5] Syso A.I, Lebedeva M.A, Khudyaev S.A, Cherevko A.S, Shishin A.I, Sebezhko O.I, Konovalova T.V, Koritkevich O.S, Petukhov V.L, Kamaldinov E.V, Slobozhanin D.M (2017). Macro-and microelements in soils and forage grasses of farm fields of the Barnaul Ob region. Bull. NSAU.

[6] Presnyak A.R (2014). Balanced Mineral Nutrition-one of the Ways to Increase Productivity in Animals. Collection of Scientific Papers of the North Caucasus Scientific Research Institute of Animal Husbandry.

[7] Kleimenova K.A (2022). Mineral Additives in Animal Feed and Their Role in Nutrition. Scientific Research of Students in Solving Urgent Problems of the Agro-Industrial Complex.

[8] Ahmad S.A, Das S.S, Khatoon A, Ansari M.T, Afzal M, Hasnain M.S, Nayak A.K (2020). Bactericidal activity of silver nanoparticles:A mechanistic review. Mater. Sci. Energy Technol.

[9] Sikora P, Augustyniak A, Cendrowski K, Nawrotek P, Mijowska E (2018). Antimicrobial activity of Al_2_O_3_, CuO, Fe_3_O_4_, and ZnO nanoparticles in scope of their further application in cement-based building materials. Nanomaterials (Basel).

[10] Marappan G, Beulah P, Kumar R.D, Muthuvel S, Govindasamy P (2017). Role of nanoparticles in animal and poultry nutrition:Modes of action and applications in formulating feed additives and food processing. Int. J. Pharmacol.

[11] Kumar H, Venkatesh N, Bhowmik H, Kuila A (2018). Metallic nanoparticle:A review. Biomed. J. Sci. Tech. Res.

[12] Parvez S, Venkataraman C, Mukherji S (2006). A review on advantages of implementing luminescence inhibition test (*Vibrio fischeri*) for acute toxicity prediction of chemicals. Environ. Int.

[13] Sizova E, Miroshnikov S, Yausheva E, Kosyan D (2015). Comparative characteristic of toxicity of nanoparticles using the test of bacterial bioluminescence. Biosci. Biotechnol. Res. Asia.

[14] Deryabin D.G, Vasil'chenko A.S, Nikiyan A.N (2011). Investigation of ampicillin effect on the morphological and mechanical properties of *Escherichia coli* and *Bacillus cereus* cells with atomic force microscopy. Antibiot. Khemother.

[15] Zhang X, Saravanakumar K, Sathiyaseelan A, Wang M.H (2022). Biosynthesis, characterization, antibacterial activities of manganese nanoparticles using *Arcopilus globulus* and their efficiency in degradation of bisphenol A. Inorg. Chem. Commun.

[16] Kaweeteerawat C, Ivask A, Liu R, Zhang H, Chang C.H, Low-Kam C, Fischer H, Ji Z, Pokhrel S, Cohen Y, Telesca D, Zink J, Mädler L, Holden P.A, Nel A, Godwin H (2015). Toxicity of metal oxide nanoparticles in *Escherichia coli* correlates with conduction band and hydration energies. Environ. Sci. Technol.

[17] Phan D.C, Vazquez-Munoz R, Matta A, Kapoor V (2020). Short-term effects of Mn_2_O_3_ nanoparticles on physiological activities and gene expression of nitrifying bacteria under low and high dissolved oxygen conditions. Chemosphere.

[18] Kolesnikov S.I, Varduni V.M, Timoshenko A.N, Denisova T.V, Kazeev K.S, Akimenko Y.V (2020). Estimation of ecotoxicity of nanoparticles of cobalt, copper, nickel and zinc oxides on biological indicators of the state of ordinary chernozem. S Russ. Ecol. Dev.

[19] Zafar B, Shafqat S.S, Zafar M.N, Haider S, Sumrra S.H, Zubair M, Akhtar M.S (2022). NaHCO_3_ assisted multifunctional Co_3_O_4_, CuO and Mn_2_O_3_ nanoparticles for tartrazine removal from synthetic wastewater and biological activities. Mater. Today Commun.

[20] Otero-González L, García-Saucedo C, Field J.A, Sierra-Álvarez R (2013). Toxicity of TiO_2_, ZrO_2_, Fe0, Fe_2_O_3_, and Mn_2_O_3_ nanoparticles to the yeast, *Saccharomyces cerevisiae*. Chemosphere.

[21] Tavakoli P, Ghaffarifar F, Delavari H, Shahpari N (2019). Efficacy of manganese oxide (Mn_2_O_3_) nanoparticles against *Leishmania major in vitro* and *in vivo*. J. Trace Elem. Med. Biol.

[22] Dalir Ghaffari A, KarimiPourSaryazdi A, Barati M, KarimiPourSaryazdi Y (2020). Evaluation of the effect of manganese oxide nanoparticles on *Toxoplasma gondii in vitro*. J. Nurse Physician War.

[23] Buchman J.T, Hudson-Smith N.V, Landy K.M, Haynes C.L (2019). Understanding nanoparticle toxicity mechanisms to inform redesign strategies to reduce environmental impact. Acc. Chem. Res.

[24] Wang D, Lin Z, Wang T, Yao Z, Qin M, Zheng S, Lu W (2016). Where does the toxicity of metal oxide nanoparticles come from:The nanoparticles, the ions, or a combination of both?. J. Hazard. Mater.

[25] Aruoja V, Pokhrel S, Sihtmäe M, Mortimer M, Mädler L, Kahru A (2015). Toxicity of 12 metal-based nanoparticles to algae, bacteria and protozoa. Environ. Sci. Nano.

[26] Sadiq I.M, Pakrashi S, Chandrasekaran N, Mukherjee A (2011). Studies on toxicity of aluminum oxide (Al_2_O_3_) nanoparticles to microalgae species:*Scenedesmus sp.* and *Chlorella sp*. J. Nanopart. Res.

[27] Rogers N.J, Franklin N.M, Apte S.C, Batley G.E, Angel B.M, Lead J.R, Baalousha M (2010). Physico-chemical behaviour and algal toxicity of nanoparticulate CeO_2_ in freshwater. Environ. Chem.

[28] Hoseinzadeh E, Makhdoumi P, Taha P, Hossini H, Stelling J, Amjad Kamal M, Md Ashraf G (2017). A review on nano-antimicrobials:Metal nanoparticles, methods and mechanisms. Curr. Drug Metab.

[29] Hedberg Y.S, Pradhan S, Cappellini F, Karlsson M.E, Blomberg E, Karlsson H.L, Wallinder I.O, Hedberg J.F (2016). Electrochemical surface oxide characteristics of metal nanoparticles (Mn, Cu and Al) and the relation to toxicity. Electrochim. Acta.

[30] Titma T, Shimmo R, Siigur J, Kahru A (2016). Toxicity of antimony, copper, cobalt, manganese, titanium and zinc oxide nanoparticles for the alveolar and intestinal epithelial barrier cells *in vitro*. Cytotechnology.

[31] Mohamed R.A, Kassem L.M, Ghazali N.M, Elgazzar E, Mostafa W.A (2023). Modulation of the morphological architecture of Mn_2_O_3_ nanoparticles to MnCoO nanoflakes by loading Co^3+^ via a co-precipitation approach for mosquitocidal development. Micromachines.

[32] Kainat Khan M.A, Ali F, Faisal S, Rizwan M, Hussain Z, Zaman N, Afsheen Z, Uddin M.N, Bibi N (2021). Exploring the therapeutic potential of *Hibiscus rosa sinensis* synthesized cobalt oxide (Co_3_O_4_-NPs) and magnesium oxide nanoparticles (MgO-NPs). Saudi J. Biol. Sci.

[33] Singh S.P, Kumari M, Kumari S.I, Rahman M.F, Mahboob M, Grover P (2013). Toxicity assessment of manganese oxide micro and nanoparticles in Wistar rats after 28 days of repeated oral exposure. J. Appl. Toxicol.

[34] Máté Z, Horváth E, Kozma G, Simon T, Kónya Z, Paulik E, Szabó A (2016). Size-dependent toxicity differences of intratracheally instilled manganese oxide nanoparticles:Conclusions of a subacute animal experiment. Biol. Trace Elem. Res.

[35] Minigalieva I, Bushueva T, Fröhlich E, Meindl C, Öhlinger K, Panov V, Varaksin A, Shur V, Shishkina E, Gurviсh V, Katsnelson B (2017). Are *in vivo* and *in vitro* assessments of comparative and combined toxicity of the same metallic nanoparticles compatible, or contradictory, or both?A juxtaposition of data obtained in respective experiments with NiO and Mn_3_O_4_ nanoparticles. Food Chem. Toxicol.

[36] Katsnelson B.A, Minigaliyeva I.A, Panov V.G, Privalova L.I, Varaksin A.N, Gurvich V.B, Sutunkova M.P, Shur V.Y, Shishkina E.V, Valamina I.E, Makeyev O.H (2015). Some patterns of metallic nanoparticles'combined subchronic toxicity as exemplified by a combination of nickel and manganese oxide nanoparticles. Food Chem. Toxicol.

[37] Yousefalizadegan N, Mousavi Z, Rastegar T, Razavi Y, Najafizadeh P (2019). Reproductive toxicity of manganese dioxide in forms of micro-and nanoparticles in male rats. Int. J. Reprod. Biomed.

[38] Horev-Azaria L, Kirkpatrick C.J, Korenstein R, Marche P.N, Maimon O, Ponti J, Romano R, Rossi F, Golla-Schindler U, Sommer D, Uboldi C, Unger R.E, Villiers C (2011). Predictive toxicology of cobalt nanoparticles and ions:Comparative *in vitro* study of different cellular models using methods of knowledge discovery from data. Toxicol. Sci.

[39] Vales G, Demir E, Kaya B, Creus A, Marcos R (2013). Genotoxicity of cobalt nanoparticles and ions in *Drosophila*. Nanotoxicology.

[40] Heinlaan M, Muna M, Juganson K, Oriekhova O, Stoll S, Kahru A, Slaveykova V.I (2017). Exposure to sublethal concentrations of Co_3_O_4_ and Mn_2_O_3_ nanoparticles induced elevated metal body burden in *Daphnia magna*. Aquat. Toxicol.

[41] Iravani S, Korbekandi H, Mirmohammadi S.V, Zolfaghari B (2014). Synthesis of silver nanoparticles:Chemical, physical and biological methods. Res. Pharm. Sci.

[42] Huang S, Wang L, Liu L, Hou Y, Li L (2015). Nanotechnology in agriculture, livestock, and aquaculture in China. A review. Agron. Sustain. Dev.

[43] Al-Beitawi N.A, Momani Shaker M, El-Shuraydeh K.N, Bláha J (2017). Effect of nanoclay minerals on growth performance, internal organs and blood biochemistry of broiler chickens compared to vaccines and antibiotics. J. Appl. Anim. Res.

[44] Hashem N.M, El-Desoky N, Hosny N.S, Shehata M.G (2020). Gastrointestinal microflora homeostasis, immunity and growth performance of rabbits supplemented with innovative non-encapsulated or encapsulated synbiotic. Proceedings.

[45] Abd El-Hack M.E, Alagawany M, Farag M.R, Arif M, Emam M, Dhama K, Sayab M (2017). Nutritional and pharmaceutical applications of nanotechnology:Trends and advances. Int. J. Pharmacol.

[46] Jahanbin R, Yazdanshenas P, Rahimi M, Hajarizadeh A, Tvrda E, Nazari S.A, Ghanem N (2021). *In vivo* and *in vitro* evaluation of bull semen processed with zinc (Zn) nanoparticles. Biol. Trace Elem. Res.

[47] Wang L, Hu C, Shao L (2017). The antimicrobial activity of nanoparticles:Present situation and prospects for the future. Int. J. Nanomedicine.

[48] Fesseha H, Degu T, Getachew Y (2020). Nanotechnology and its application in animal production:A review. Open J. Vet. Med.

